# Prevalence, knowledge and attitudes toward herbal medication use by Saudi women in the central region during pregnancy, during labor and after delivery

**DOI:** 10.1186/s12906-017-1714-3

**Published:** 2017-04-04

**Authors:** Sameer Al-Ghamdi, Khaled Aldossari, Jamaan Al-Zahrani, Fawaz Al-Shaalan, Saad Al-Sharif, Hamad Al-Khurayji, Aiman Al-Swayeh

**Affiliations:** 1 Department of Family Medicine, College of Medicine, Prince Sattam bin Abdulaziz University, Al Kharj, Saudi Arabia; 2 College of Medicine, Prince Sattam bin Abdulaziz University, Al Kharj, Saudi Arabia

**Keywords:** Herbal medicine, Prevalence, Pregnancy, Saudi Arabia

## Abstract

**Background:**

Herbal medication usage is prevalent in both developing and developed countries. The low level of awareness of the possible dangers of some herbs during pregnancy increases the risk of unwarranted sequelae. This manuscript describes the first study of herbal medication use among pregnant women in Saudi Arabia. It aims to determine the prevalence of herbal medication use during pregnancy, during labor and after delivery in the central region of Saudi Arabia.

**Methods:**

A cross-sectional descriptive study was conducted over a 5-month period from May 15 to October 15, 2016. A self-administered questionnaire was distributed at 4 main hospitals and 3 primary care centers in Riyadh and Al Kharj. Data from 612 participants were collected and analyzed. Descriptive statistics in the form of frequency and percentage were determined, and Chi-squared tests were performed.

**Results:**

Of the 612 participants, 25.3%, 33.7% and 48.9% used herbs during pregnancy, during labor, and after delivery, respectively. The primary motives for using herbal medication during pregnancy, during labor and after delivery were to boost general health, ease and accelerate labor and clean the womb, respectively. There was a significant association between use during pregnancy and prior use (*P* = 0.001). Most pregnant women used herbs based on advice from family and friends (52.9%). Only 40.7% of pregnant women disclosed their herbal use to their doctors.

**Conclusion:**

The prevalence of herbal medication use among pregnant Saudi women in Riyadh and Al Kharj is relatively high. Doctors should be aware of evidence regarding the potential benefits or harm of herbal medication use during pregnancy.

## Background

Herbal medicine refers to using the seeds, berries, roots, leaves, bark, or flowers of a plant for medicinal purposes [1]. The most widely used herbs include garlic, St. John’s wort, ginger, ginseng, Echinacea, kava and devil’s claw [2, 3]. These herbs are commonly used by pregnant women to relieve various complaints they experience during pregnancy. For example, ginger is used to relieve the nausea associated with pregnancy, whereas devil’s claw and St John’s wort are used to relieve back pain and depression [4]. Additionally, herbs are commonly used to induce labor, with the most frequently used herbs including castor oil, black and blue cohosh and red raspberry leaf [5].

The use of herbal medications is prevalent in both developing and developed countries. In China, herbal medicine accounts for approximately 50% of total medicine consumption [6]. A review study conducted by Hall et al. found that herbal medication use during pregnancy in several countries, including the United States, Australia, Sweden, Norway, Italy and Hong Kong, ranges from 1% to 87% [7].

In the Middle East, herbal medication use during pregnancy is fairly common. A review study that investigated this practice in nine Middle Eastern countries found that the prevalence of herbal medication use during pregnancy ranged from 22.3% to 82.3% and that the most commonly used herbs included peppermint, ginger, thyme chamomile, sage, aniseeds, fenugreek, green tea, and garlic [3].

Biomedicine has replaced herbal medicine in many applications. However, many woman still use herbs during pregnancy to relieve discomfort because many women believe that herbs cause less harm than biomedicine [3, 8, 9]. This can be attributed to the fact that many biomedical drugs and procedures have documented adverse effects on fetal development. For example, certain imaging techniques, such X-rays, have demonstrated the devastating effect of certain drugs, such as diethylstilbestrol, certain chemotherapeutics and even some antibiotics on pregnancy outcome [10]. This evidence coupled with the fact that most physicians warn pregnant women regarding the use of any biomedical drugs without recommendation from their physician encourages women to seek an alternative method.

Although many herbs are considered safe during pregnancy, some are not [11]. Multiple case report studies have linked the use of certain herbal medications with adverse events in the infant. For example, black and blue cohosh, which are used to induce uterine contractions, have been linked to cases in which the infants experienced myocardial infarctions, heart failure, seizures and kidney damage [12, 13]. Another study linked the use of Tripterygium wilfordii, which is used for rheumatoid arthritis and other indications, to an infant with occipital meningoencephalocele and cerebellar agenesis [14]. Additionally, licorice, which is used as a natural remedy and a sweetener in candies, liquor, and teas, might be linked to an increase in the risk of still births, miscarriages and lower gestational age at birth [5, 15, 16].

The low awareness about the possible dangers of some herbs during the pregnancy period combined with the fact that natural herbs and vitamin supplements are not subjected to the FDA evaluation process required for prescription drugs increases the risk of unwarranted sequelae [17].

Little information is available about the use of herbs during pregnancy in Saudi Arabia, and no previous studies about herbal medication use among pregnant women have been conducted in Saudi Arabia.

The primary purposes of this study are to determine the prevalence, motives behind, and degree of disclosure of herbal medicine use during pregnancy, during labor and after delivery in the central region of Saudi Arabia.

## Methods

A cross-sectional descriptive study was conducted over a 5-month period from May 15 to October 15, 2016. The study was conducted at three main hospitals in Riyadh: King Faisal Specialist Hospital, King Khalid University Hospital and Al Iman General Hospital. The study was also conducted at the Military Hospital and three primary health care centers in Al Kharj city. The inclusion criteria were as follows: Saudi women in the central region who had previously completed a successful pregnancy, who were at least 18 years of age, and whose last pregnancy was less than 3 years prior. The exclusion criteria were as follows: women who were pregnant for the first time and had not yet experienced labor and delivery, who were younger than 18 years of age, and whose last pregnancy was more than 3 years prior (to minimize recall bias). The sample size was estimated according to the following formula: N = (Zα)^2^ × ([*p*(1-*p*)]/d^2^), where n is the estimated sample size, Zα at the 5% level of significance equals 1.96, d is the level of precision and was estimated to equal 0.05, and p is the prevalence rate of complementary and alternative medicine use determined by two previous studies conducted in the region (approximately 30%). Hence, the primary sample size is [(1.96)^2^ × (0.3 × 0.7)]/(0.05 × 0.05), which equals 323 subjects. The actual sample size is the primary sample size × design effect (estimated as 1.5), which yields 484 subjects. The expected response rate is estimated to be 80%. Therefore, the planned sample size was 484 × 100 / 80, which equals 606 subjects.

A validated, confidential, self-administered, semi-structured questionnaire was used as the data collection tool. The questionnaire has been used in a previous study at Moi University, Kenya [18]. Permission to use the questionnaire was obtained through e-mail. The questionnaire contained 42 questions, with 36 close-ended questions and 5 open-ended questions. Of the 36 open-ended questions, 11 contained the option “other”. The questionnaire also contained definitions of both herbal medicine and prescription medicine at the beginning of the questionnaire to rule out any confusion that may arise during the filling process.

The questionnaire was translated into Arabic and then back-translated to English by two Family Medicine consultants fluent in Arabic. A pilot test including 20 participants was performed prior to the distribution of the questionnaire. No changes were made after the pilot test because none of the participants faced any problems answering any of the questions.

Letters of authorization were obtained from each hospital prior to the distribution of the questionnaire. The questionnaire was distributed in the waiting rooms of the outpatient departments of the previously mentioned hospitals by well-trained staff, and verbal informed consent was obtained from the participants. The proposal was approved by the Ethics Committee of Prince Sattam bin Abdulaziz University Institutional Review Board with IRB Number PSAU-2015-IM/12–10/PI. Hard copies of the translated questionnaire were distributed at three main hospitals in Riyadh: 170 questionnaires collected from King Khalid university hospital, 149 questionnaires from King Faisal specialist hospital, and 143 questionnaires from Al Iman general hospital.

**Fig. 1:**
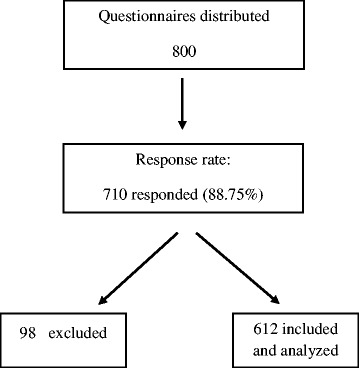
Flow chart showing the data collection process

The questionnaire was also distributed at the military hospital and three primary health care centers in Al Kharj city, which collected 178 and 160 questionnaires, respectively. Different hospitals and primary health care centers were selected to ensure that all different populations were represented and to minimize bias. For example, a military hospital only serves the families of military personnel. The flow chart shows the distribution process with the response rate (as shown in Fig. 1).

A non-randomized convenience sampling method was used during the distribution of the questionnaire.

### Data entry and analysis

After excluding incomplete questionnaires, which were defined as any questionnaire with missing responses to more than 5 questions, data from 612 participants were obtained and analyzed using the Statistical Package for Social Sciences v22. Both analysis of descriptive statistics in the form of frequency and percentage and the chi-squared test were performed. *P* values equal to or less than 0.05 were considered statistically significant.

## Results

### Sociodemographic characteristics of the participants

Table 1 shows that the majority of participants (44.8%) had at least four children, 90.8% were married, most had a bachelor’s degree (41.2%), only a few (4.4%) had no formal education, 49.2% were housewives, 45.1% lived closer than 5 km from the closest health facility, and the majority (80.1%) had at least three antenatal care visits.

**Table 1:** Sociodemographic characteristics of the study participants

		Number	Percentage
Children	One	116	19%
	Two	108	17.6%
	Three	114	18.6%
	Four or more	274	44.8%
Marital status	Married	556	90.8%
	Divorced	31	5.1%
	Widowed	25	4.1%
Education level	No formal education	27	4.4%
	Primary education	33	5.4%
	Secondary education	52	8.5%
	High school	148	24.2%
	Diploma	82	13.4%
	Bachelor’s degree	252	41.2%
	Master’s degree and higher	18	2.9%
Occupation	Housewife	301	49.2%
	Private sector employee	48	7.8%
	Government employee	243	39.7%
	Other	20	3.3%
Closest health facility	Not far (<5 km)	276	45.1%
	Somewhat far (5 km – 10 km)	218	35.6%
	Very far (>10 km)	118	19.3%
Antenatal care visits	Zero	24	3.9%
	One or two	98	16%
	Three or more	490	80.1%

### Prevalence of herbal medication use during pregnancy, during labor and after delivery and its association with the socio-demographic characteristics of the participants

**Fig. 2:**
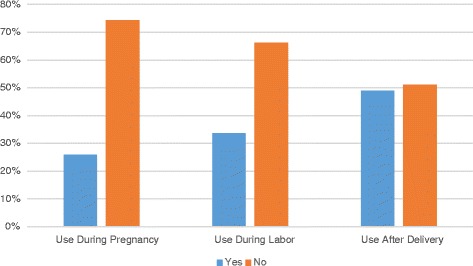
Prevalence of herbal medicine use during pregnancy, during labor and after delivery

The prevalence of herbal medication use during pregnancy was 25.3%, while 33.7% of the participants used herbs during labor and 48.9% used them after delivery (Fig. 2). The search for an association between herbal use during pregnancy and the sociodemographic characteristics of the participants indicated an insignificant association regarding number of children, marital status, education level and the distance from nearest health facilities (Table 2). However, there was a significant association between herbal medication use and the number of antenatal care visits (*P* = 0.031), with a higher usage level among women who visited antenatal clinics regularly (Table 2).

**Table 2:** Association between herbal medication use during pregnancy and sociodemographic characteristics

		Number	Herbal medication use		Chi squared	*P* value
			Yes	No		
			N (%)	N (%)		
Children	One	114	22 (19.3%)	92 (80.7%)	3.789	0.285
	Two	108	31 (27.9%)	80 (72.1%)		
	Three	111	27 (24.1%)	85 (75.9%)		
	Four or more	268	75 (28%)	193(72%)		
Marital status	Married	546	142 (26%)	404 (74%)	0.188	0.910
	Divorced	31	7 (22.6%)	24 (77.4%)		
	Widowed	24	6 (25%)	18 (75%)		
Education level	No formal education	27	7 (25.9%)	20 (74.1%)	2.868	0.825
	Primary education	32	9 (28.1%)	23 (71.9%)		
	Secondary education	51	16 (31.4%)	35 (68.6%)		
	High school	144	39 (27.1%)	105 (72.9%)		
	Diploma	81	23 (28.4%)	58 (71.6%)		
	Bachelor’s degree	249	56 (22.5%)	193 (77.5%)		
	Master’s degree and higher	17	5 (29.4%)	12(70.6%)		
Occupation	Housewife	296	74 (25%)	222 (75%)	1.298	0.730
	Private sector employee	44	14 (31.8%)	30 (68.2%)		
	Government employee	241	63 (26.1%)	178 (73.9%)		
	Other	20	4 (20.0%)	16 (80.0%)		
Closest health facility	Not far (<5 km)	270	66 (24.4%)	204(75.6%)	0.849	0.654
	Somewhat far (5 km – 10 km)	217	56 (25.8%)	161(74.2%)		
	Very far (>10 km)	114	33 (28.9%)	81 (71.1%)		
Antenatal care visits	Zero	49	5 (10.2%)	44 (89.8%)	6.977	0.031
	One or two	91	23 (25.3%)	68 (74.7%)		
	Three or more	461	127 (27.5%)	334(72.5%)		

### Motives behind using herbal medicine during pregnancy, during labor and after delivery

During pregnancy, 77.2% of participants reported using medical herbs to boost and maintain health despite being healthy during labor, and the majority (58%) used herbs to ease and accelerate labor. The most prevalent motives behind using herbs after delivery where to clean the womb (33.2%), to relieve pain (25.1%) and to improve general health (24.7%) (Table 3).

**Table 3:** Motives behind using herbal medicine during pregnancy, during labor and after delivery

Period	Purpose	Percentage
During pregnancy	I was healthy and wanted to boost or maintain my health	77.2%
	I was ill and I wanted to relieve or cure ailment/illness	22.8%
During labor	To induce labor	21.3%
	To ease or speed up labor	58.0%
	Bleeding-related use	12.2%
	Fetus-related use	5.3%
	Other	3.2%
After delivery	General health	24.7%
	Relieve pain	25.1%
	To clean the womb	33.2%
	To decrease bleeding	2.8%
	To increase lactation	6.5%
	To lose weight	7.7%

### Health-seeking behavior

Of the 155 women who used herbs during pregnancy, 75.4% used herbs prior to pregnancy. Table 4 shows a significant association between herbal medication use during pregnancy and prior use, with 117 (30.7%) of 381 women who had ever used herbal medication using it also during pregnancy (*P* = 0.001).

**Table 4:** Association between herbal medication use during pregnancy and ever use

		Did you use herbal medicine at any point during pregnancy?		Chi squared	*P* value
		Yes	No		
Have you ever used herbal medicine unrelated to pregnancy?	Yes	117	264	11.331	0.001
	No	38	173		

**Fig. 3:**
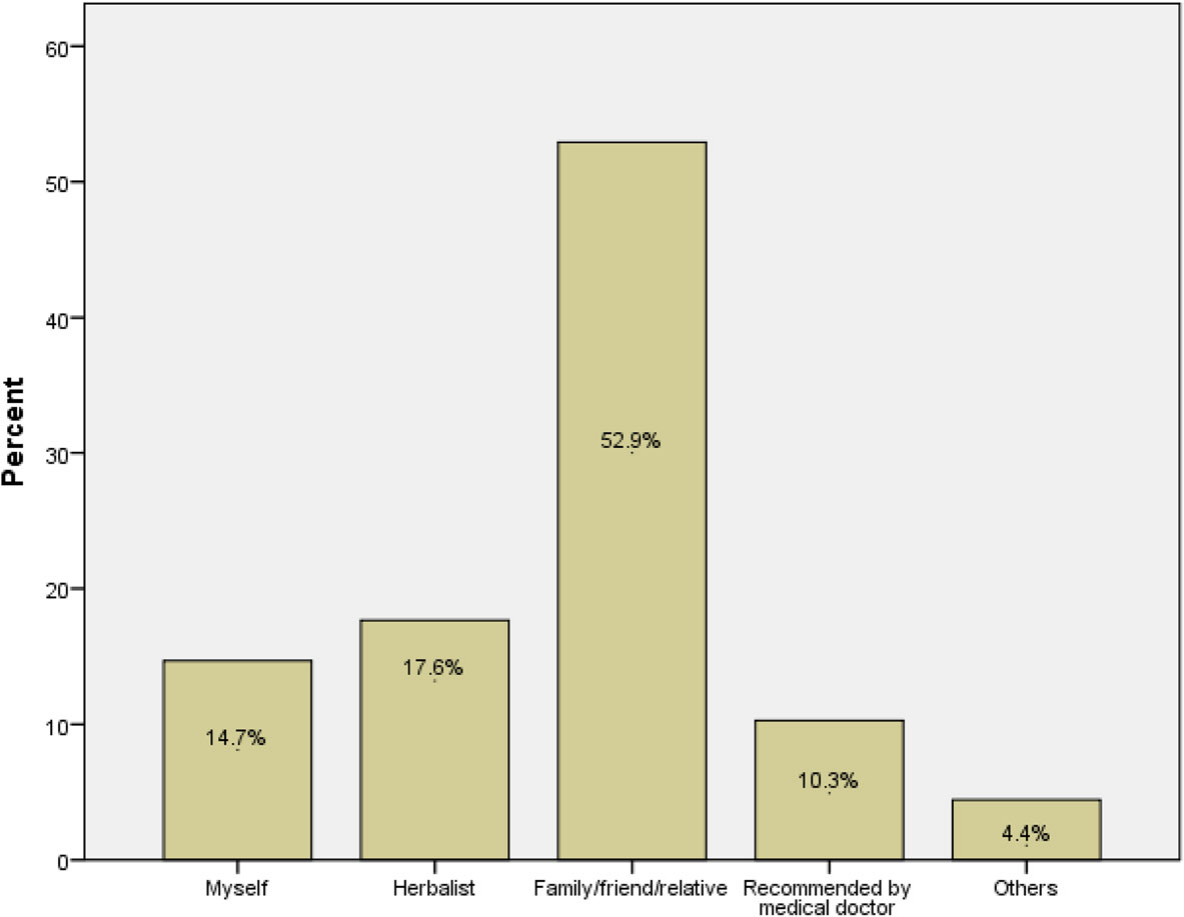
Prescription source of herbal medicine during pregnancy

The most common source of herbal medication was herbal shops (86.5%), and most participants (52.9%) used herbal medication based on advice from family, friends or relatives (Fig. 3).

### Disclosure of herbal medication use

**Fig. 4:**
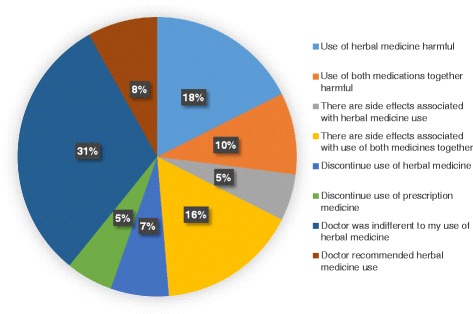
Doctor’s advice regarding herbal medication use during pregnancy

Only 40.7% of pregnant women disclosed their herbal medication use to their doctors. When the usage was disclosed, the majority (31.1%) reported that the doctor was indifferent about the use of herbal medication (Fig. 4).

### Participants’ thoughts and beliefs about the use of herbal and biomedicines

The majority of participants believe that most herbal and prescription medications are not safe during pregnancy.

There is uncertainty about the safety of herbal medication for general use. However, the majority believe that herbal medication can be useful if a doctor or an herbalist recommended its use (Table 5).

**Table 5:** Participants’ thoughts about the safety of herbal and conventional medications

	Agree (%)	Disagree (%)	Not sure (%)
Most Western medicine is not safe for me (mother) during pregnancy	66.2	18.9	14.9
Most Western medicine is not safe for my baby during pregnancy	68.3	17.7	13.9
Most herbal medicine is not safe for me during pregnancy	67.3	15.6	17.1
Most herbal medicine is not safe for my baby during pregnancy	68.7	13.2	18.1
Most herbal medicine is natural	45	22.9	31.9
Most herbal medicine is safe	25.3	38.9	35.8
Herbals are beneficial if recommended by doctor	62.5	18.5	19
Herbals are beneficial if recommended by herbalist	35.6	34.1	30.2
Herbals are beneficial if recommended by family/relative	19.6	49.8	30.6
There are illnesses or conditions for which herbal medicine is more effective than Western medicine	54.6	19.7	25.7
There are illnesses or conditions for which Western medicine is more effective than herbal medicine	65.4	10.2	24.4

## Discussion

Despite the improved accessibility to healthcare services and the higher level of education that women enjoy in in Riyadh and Al Kharj, herbal medication use during pregnancy is common (25.3%) in these areas compared to the usage reported in other studies. For example, the study conducted by Mothupi [18], which was a nonrandom convenience sample study performed in an urban area with lower accessibility to healthcare services and a lower level of education, revealed that only 12% of the participants used herbal medication during pregnancy. Furthermore, this high percentage confirms the general trend toward herbal medication use during pregnancy, as shown in multiple studies conducted worldwide [19–23].

It is commonly believed that less educated women are more likely to use herbal medication; however, our study demonstrated otherwise. In contrast to the findings of Mothupi [18], which indicated that the majority of the users had an education level of high school or uneducated (79.2%), the majority of the participants in our study (57.5%) had an education level greater than high school (Diploma, Bachelor degree or higher). Additionally, only 8.7% of the participants whose education level was greater than high school in the study by Mothupi [18] reported using herbs during pregnancy, whereas 24.2% of the participants who had an education level greater than high school in our study declared using herbs during pregnancy. A similar difference was found in the study conducted by Titilayo O Fakeye, Rasaq Adisa and Ismail E Musa in Nigeria, where 68.3% of the participants had an education level of high school down to uneducated [24].

One of the primary aims of this study was to determine the degree of disclosure between herbal medicine users and their doctors. The results were alarming because only 40.7% of pregnant women disclosed herbal medication use with their doctors. In particular, our results revealed a higher usage level among women who visited antenatal clinics regularly. When the usage was disclosed, the majority (31.1%) reported that the doctor was indifferent about the use of herbal medication. Herbal shops were the most common source (86.5%). Multiple studies have shown that most pregnant women use herbal medications based on advice from their family and friends. Our study shows the same result, with more than half of the participants using herbal medications based on advice from family and friends [23–26].

Of the 155 women who used herbs during pregnancy, 75.4% used herbs prior to pregnancy; 117 (30.7%) of 381 women who had any prior use of herbal medication also used herbal medication during pregnancy. These results are similar to those found by Mothupi [18], who showed that 26.8% of those who used herbal medicine before pregnancy also used it during pregnancy.

## Limitations

The main limitation of this study was that the participants were recruited through a nonrandom convenience sampling method from 4 hospitals and 3 primary care centers in 2 cities; as a result, the results cannot be generalized to the whole Saudi population. Further studies across Saudi Arabia, including rural and low income areas, are needed to identify the extent of this practice in Saudi Arabia.

The other limitation is that the questionnaire did not include questions regarding the specific herbs used, their dosage and the administration route during pregnancy. These questions were considered to be beyond the scope of this study because the main aims were to determine the prevalence, knowledge and attitudes toward herbal use during pregnancy.

## Recommendations

- Further studies should be conducted to determine the prevalence and pattern of herbal medication use during pregnancy, especially in low income and rural areas.
- Awareness should be raised about the possible side effects of herbal medication use.
- Health care providers should be aware of evidence regarding the potential benefits or harm of herbal medication use during pregnancy and should actively ask about herbal medication use.
- Prospective studies should be conducted on the long-term effects of commonly used herbs.

## Conclusion

Herbal medication is commonly used during pregnancy by Saudi women. Of the 612 participants, 25.3% used herbs during pregnancy, 33.7% used it during labor and 48.9% used it after delivery. The primary motives behind using herbal medication during pregnancy, during labor and after delivery where to boost general health, ease and accelerate labor and clean the womb, respectively. There was a significant association between use during pregnancy and prior use (*P* = 0.001). Most pregnant women used herbal medications based on advice from family and friends (52.9%), and herbal shops were the most common source (86.5%). Only 40.7% of pregnant women disclosed the herbal medication use to their doctors. There is uncertainty about the safety of herbal medication for general use.

## References

[1] University of Maryland medical center. Herbal medicine. 2015. https://umm.edu/health/medical/altmed/treatment/herbal-medicine. Accessed 24 Oct 2015.

[2] Holst L, Nordeng H, Haavik S (2008). Use of herbal drugs during early pregnancy in relation to maternal characteristics and pregnancy outcome. Drug Saf.

[3] John LJ, Shantakumari N (2015). Herbal medicines use during pregnancy: a review from the Middle East. Oman Med J.

[4] Ernst E, Pittler MH, Stevinson C, White AR, Eisenberg D (2001). The desktop guide to complementary and alternative medicine.

[5] McFarlin BL, Gibson MH, O’Rear J, Harman P (1999). A national survey of herbal preparation use by nurse-midwives for labor stimulation. Review of the literature and recommendations for practice. J Nursemidwif.

[6] World Health Organization. Traditional medicine. Fact sheet No. 134. 2003. http://www.who.int/mediacentre/factsheets/2003/fs134/en/. Accessed 24 Oct 2015.

[7] Hall HG, Griffiths DL, Mckenna LG (2011). The use of complementary and alternative medicine by pregnant women: a literature review. Midwifery.

[8] Westfall RE (2003). Herbal healing in pregnancy: women's experiences. J Herb Pharmacother.

[9] Bacchini M, Cuzzolin L, Camerlengo T, Velo G, Benoni G (2008). Phytotherapic compounds: the consumer-pharmacist relationship. Drug Saf.

[10] Troisi R, Hatch EE, Titus-ernstoff L, Hyer M, Palmer JR, Robboy SJ (2007). Cancer risk in women prenatally exposed to diethylstilbestrol. Int J Cancer.

[11] Saper RB. Overview of herbal medicine and dietary supplements. UpToDate. 2016. http://www.uptodate.com/contents/overview-of-herbal-medicine-and-dietary-supplements?source=search_result&search=overview+of+herbal+medicine+and+dietarysupplements&selectedTitle=1~150. Accessed 24 Oct 2015.

[12] Jones TK, Lawson BM (1998). Profound neonatal congestive heart failure caused by maternal consumption of blue cohosh herbal medication. J Pediatr.

[13] Gunn TR, Wright IMR (1996). The use of black and blue cohosh in labour. N Z Med J.

[14] Takei A, Nagashima G, Suzuki R, Hokaku H, Takahashi M, Miyo T (1997). Meningoencephalocele associated with Tripterygium wilfordii treatment. Pediatr Neurosurg.

[15] Choi JS, Han JY, Ahn HK, Ryu HM, Kim MY, Chung JH (2013). Fetal and neonatal outcomes in women reporting ingestion of licorice (glycyrrhiza uralensis) during pregnancy. Planta Med.

[16] Strandberg TE, Järvenpää AL, Vanhanen H, Mckeigue PM (2001). Birth outcome in relation to licorice consumption during pregnancy. Am J Epidemiol.

[17] Skerrett PJ. FDA needs stronger rules to ensure the safety of dietary supplements. Harvard Health Publications. 2012. http://www.health.harvard.edu/blog/fda-needs-stronger-rules-to-ensure-the-safety-of-dietary-supplements-201202024182.

[18] Mothupi MC (2014). Use of herbal medicine during pregnancy among women with access to public healthcare in Nairobi, Kenya: a cross-sectional survey. BMC Complement Altern Med.

[19] Sattari M, Dilmaghanizadeh M, Hamishehkar H, Mashayekhi SO (2012). Self-reported use and attitudes regarding herbal medicine safety during pregnancy in Iran. Jundishapur J Nat Pharm Prod.

[20] Al-riyami IM, Al-busaidy IQ, Al-Zakwani IS (2011). Medication use during pregnancy in Omani women. Int J Clin Pharm.

[21] Orief YI, Farghaly NF, Ibrahim MIA (2014). Use of herbal medicines among pregnant women attending family health centers in Alexandria. Middle East Fertil Soc J.

[22] Rahman AA, Sulaiman SA, Ahmad Z, Salleh H, Daud WN, Hamid AM (2009). Women's attitude and sociodemographic characteristics influencing usage of herbal medicines during pregnancy in Tumpat District, Kelantan. Southeast Asian J Trop Med Public Health.

[23] Nordeng H, Havnen GC (2004). Use of herbal drugs in pregnancy: a survey among 400 Norwegian women. Pharmacoepidemiol Drug Saf.

[24] Adisa R, Musa IE, Fakeye TO (2009). Attitude and use of herbal medicines among pregnant women in Nigeria. BMC Complement Altern Med.

[25] Hollyer T, Boon H, Georgousis A, Smith M, Einarson A (2002). The use of CAM by women suffering from nausea and vomiting during pregnancy. BMC Complement Altern Med.

[26] Holst L, Wright D, Nordeng H, Haavik S (2009). Use of herbal preparations during pregnancy: focus group discussion among expectant mothers attending a hospital antenatal clinic in Norwich, UK. Clin Pract.

